# Relation between athletic club affiliation from school age and future serum free testosterone levels in Japan: A cross‐sectional study

**DOI:** 10.1002/hsr2.1496

**Published:** 2023-08-17

**Authors:** Yuki Kato, Kazuyoshi Shigehara, Tomomi Nakagawa, Shohei Kawaguchi, Kouji Izumi, Yoshifumi Kadono, Atsushi Mizokami

**Affiliations:** ^1^ Department of Integrative Cancer Therapy and Urology Kanazawa University Graduate School of Medical Science Kanazawa Japan; ^2^ Depatment of Urology Wajima Municipal hospital Wajima Japan

**Keywords:** athletic club affiliation, hypogonadism, testosterone

## Abstract

**Background and Aims:**

Testosterone deficiency is often related to geriatric syndrome including erectile dysfunction, osteo‐porosis, depression, cognitive impairment, cardiovascular diseases and frailty. Despite the existence of many studies on short‐term exercise and serum testosterone levels, few research have focused on exercise habits from young age and testosterone values in middle‐aged male. In this study, we investigated whether belonging to an athletic club from school age could predict serum‐free testosterone (FT) levels.

**Methods:**

The subjects were 1609 middle‐aged male outpatients aged 40 years or older (median: 61 years, interquartile range: 54–69) who visited our hospital. Participants had their FT values measured in the morning hours during the period from December 2007 to June 2009. A questionnaire survey on exercise habits was conducted at the same time as the measurements. The exercise habit questionnaire was created based on whether the patients belonged to an athletic club in (a) elementary school, (b) junior high school, (c) high school, (d) college, (e) adult life, and (f) at the time of the test.

**Results:**

There was only one positive response to the questionnaire among 456 patients (28% of total), followed by zero for 358 patients (22% of total). The number of patients with low‐testosterone levels (FT < 8.5 pg/mL) according to the Japanese diagnostic criteria for late‐onset of hypogonadism was 839 (52.1%). In multivariate analysis, it was shown that with low‐testosterone levels (FT < 8.5 pg/mL), age (odds ratio [OR]: 1.065, 95% confidence interval [CI]: 1.052–1.079; *p* < 0.001), hypertension (OR: 3.489, 95% CI: 2.728–4.462; *p* < 0.001), type‐2 diabetes (OR: 3.035, 95% CI: 2.296–4.01; *p* < 0.001), and dyslipidemia (OR: 2.039, 95% CI: 1.558–2.668; *p* < 0.001) were risk factors, and more than two positive responses to the questionnaire (OR: 0.886, 95% CI: 0.802–0.980; *p* = 0.018) were also a significant independent factor.

**Conclusion:**

A sports club membership during school years may affect future testosterone levels.

## 1 INTRODUCTION

Serum testosterone levels in men decrease with aging.^1^ Furthermore, a previous study elucidated the existence of a significant inverse correlation between age and free testosterone (FT) levels among Japanese men.^2^ Testosterone deficiency is often related to geriatric syndrome including erectile dysfunction, osteo‐porosis, depression, cognitive impairment, and frailty.^3^ Moreover, several prospective and cross‐sectional studies revealed testosterone levels showed an inverse relationship with classical coronary risk factors, such as diabetes mellitus (DM), dyslipidemia, hypertension, and obesity.^4^, ^5^, ^6^, ^7^ These factors are components of the metabolic syndrome and have been renowned as risk factors for stroke and cardiovascular diseases (CVDs). In actual clinical practice, researchers have clarified testosterone deficiency is associated with the progression of CVDs in Caucasians and Asians.^8^, ^9^, ^10^ Therefore, there is a worldwide clinical interest in whether treatment of metabolic syndrome, which is closely related to testosterone deficiency, could prevent the development of CVDs. Multiple factors may affect testosterone levels, including aging as well as lifestyle element such as smoking, alcohol, and exercise.^11^, ^12^, ^13^ In a research examined the relevance between testosterone and physical activity, adult male raised their testosterone levels by 9% following regular aerobic exercise for 12 weeks.^14^

Despite the existence of many studies on short‐term exercise and serum testosterone levels, few research have focused on exercise habits from young age and testosterone values in middle‐aged male. Therefore, this research aimed to investigate the relevance between testosterone value and exercise habits from school age to adolescence based on questionnaire surveys.

## 2 MATERIALS AND METHODS

### 2.1 Study approval and ethical considerations

We published a written explanation of this retrospective study on our hospital website and in the outpatient clinic, so that patients could refuse to participate in this research. The study, including the method of obtaining consent mentioned above, was approved by the Kanazawa University Hospital Ethics Committee (approval no. 2021‐226).

### 2.2 Study population

From 2007 to 2009, 1682 male subjects aged more than 40 years with metabolic factors, comprising, dyslipidemia, hypertension, and type‐2 DM, inspected serum FT value at our outpatient clinic for the testing late‐onset hypogonadism (LOH) syndrome. At the first visit, blood was collected between 09:00 and 11:00 to determine serum FT values employing radioimmunoassay (DPC Free Testosterone Kit, Mitsubishi Kagaku Iatron). All 1682 of these patients were included as inclusion criteria for our study. The diagnosis of hypogonadism was based on the Japanese criteria for FT levels ≤8.5 pg/mL.^15^ Dyslipidemia, type‐2 DM, and hypertension were diagnosed in compliance with the Japanese diagnostic criteria or medical treatment with any agent for such diseases.^16^, ^17^ A total of 73 patients who did not fill the questionnaire were eliminated. No further inclusion and exclusion criteria were established to analyze real data from actual clinical practice. Among the 1682 patients, 1609 were eligible for the analysis.

### 2.3 Questionnaire on exercise habits from school age

The participants were surveyed about exercise habits from school age based on a questionnaire that was completed before the physical examination. The exercise habit questionnaire was created based on whether or not the patients belonged to an athletic club in (a) elementary school, (b) junior high school, (c) high school, (d) college, (e) adult life, and (f) at the time of the test (Supporting Information: Figure S1). The exercise patterns from childhood were assessed by the number of positive (“yes”) responses to the questionnaire.

### 2.4 Data analysis

First, the FT levels per response to the exercise habits questionnaire were compared by analysis of variance (ANOVA) test. The collected data were divided into two groups according to serum FT levels (≤8.5 pg/mL or >8.5 pg/mL). They were then compared in univariate analysis. Chi‐squared test and the Mann–Whitney *U*‐test were applied to decide the factors related to hypogonadism. The multivariate logistic regression model was made on the ground of the same covariates in the univariate analysis. The statistical analyses were conducted using SPSS™ statistics (version 22, SPSS Inc.). In all analyses, *p*‐values of <0.05 were considered statistically significant using a two‐tailed test.

## 3 RESULTS

In our study (1609 participants), the median of age and serum FT level were 61 (interquartile range [IQR]: 54–69) and 8.3 pg/mL (6.4–10.3), respectively (Table 1). No significant differences were found in FT levels per response to the questionnaire on exercise habits (Table 2). Biochemical hypogonadism was monitored in 839 cases (52.1%) on the basis of the Japanese criteria (≤8.5 pg/mL)^15^ (Table 3).

**Table 1:** Patient background information.

*N* = 1609	Median	IQR
Age	61	54–69
BMI	23.7	21.9–25.7
FT level (pg/mL) at baseline	8.3	6.4–10.3
Number of positive (“yes”) questionnaire responses	1	1–3

	*N*	%
Dyslipidemia, yes	512	31.8
Hypertension, yes	796	49.4
Type‐2 diabetes, yes	442	27.5

Abbreviations: BMI, body mass index; FT, free testosterone; IQR, interquartile range.

**Table 2:** The result between FT levels and each option.

			*p* = 0.206^a^
	*N*	Median (pg/mL)	IQR (pg/mL)
Q (a), yes	338	9.09	7.0–11.1
Q (a), no	1271	8.32	6.2–10.0
Q (b), yes	867	8.86	6.75–10.8
Q (b), no	742	8.04	6.0–9.7
Q (c), yes	643	8.69	6.6–10.6
Q (c), no	966	8.34	6.2–10.2
Q (d), yes	190	8.86	6.7–10.9
Q (d), no	1419	8.43	6.3–10.3
Q (e), yes	518	8.72	6.8–10.4
Q (e), no	1091	8.37	6.2–10.3
Q (f), yes	211	8.53	6.7–10.6
Q (f), no	1398	8.47	6.3–10.3

Abbreviations: FT, free testosterone; IQR, interquartile range; Q, the questionnaire.

^a^ Nonrepeated measures analysis of variance.

**Table 3:** Backgrounds of patients are divided into two groups according to Japanese guidelines on LOH syndrome.

*N* = 1609	FT < 8.5 pg/mL group (*N* = 839)		FT > 8.5 pg/mL group (*N* = 707)		
	Median	IQR	Median	IQR	*p*‐value
Age	66	58–73	58	50–65	**<0.01**
BMI	23.8	21.8–25.8	23.5	21.9–25.6	0.99
FT level (pg/mL) at baseline	6.4	5.2–7.5	10.4	9.3–12.1	**<0.01**
Number of positive questionnaire responses	1	1–3	2	1–3	**<0.01**

	*N*	%	*N*	%	
Dyslipidemia, yes	363		149		**<0.01**
Hypertension, yes	567		229		**<0.01**
Type‐2 diabetes, yes	339		103		**<0.01**

*Note*: Bold values indicate *p* < 0.05.

Abbreviations: BMI, body mass index; FT, free testosterone; IQR, interquartile range; LOH, late‐onset hypogonadism.

The baseline characteristics of the patients on the basis of FT levels are summarizing in Table 3. Participants with hypogonadism who were significantly older were associated with fewer positive questionnaire responses and indicated a higher frequency of developing hypertension, dyslipidemia, and type‐2 diabetes compared with those without hypogonadism. No significant difference in BMI was observed between the two groups (Table 3).

**Figure 1:**
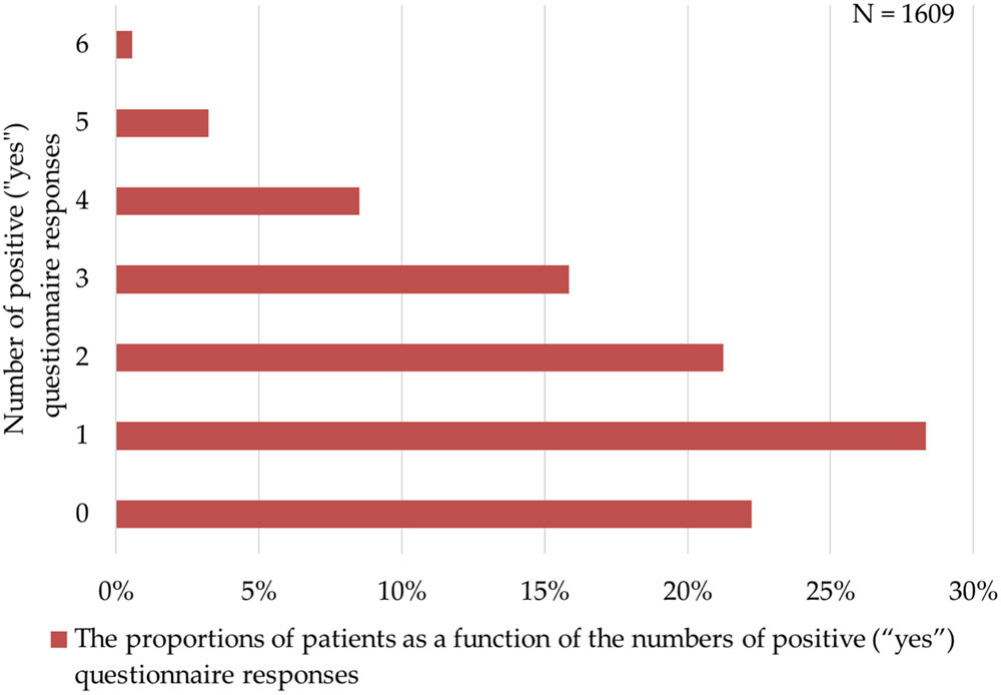
Plot showing the proportions of patients as a function of the numbers of positive (“yes”) questionnaire responses.

**Figure 2:**
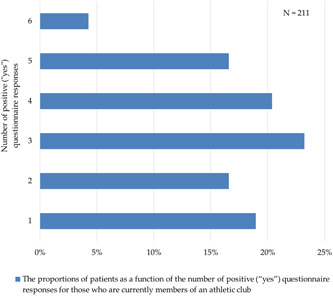
Proportions of patients as a function of the number of positive (“yes”) questionnaire responses for those who are currently members of an athletic club.

The results of the questionnaire are displayed in Figures 1 and 2. There was only one positive response to the questionnaire among 456 patients (28% of total), followed by zero for 358 patients (22% of total) (Figure 1). Those who answered that they were currently in an athletic club (211/1609) were those who most likely had three positive questionnaire responses, which corresponded to 23% (49/211), followed by 20% (43/211) for those who provided four positive responses, and 64% (136/211) for those who provided three or more positive responses (Figure 2).

On multivariate analysis, age, dyslipidemia, hypertension, and type‐2 diabetes were significantly associated with FT < 8.5 pg/mL (odds ratio [OR] = 2.039; 95% confidence interval [CI] = 1.558–2.668; *p* < 0.001, OR = 3.489; 95% CI = 2.728–4.462; *p* < 0.001, and OR = 3.035; 95% CI = 2.296–4.01; *p* < 0.001, respectively) (Table 4). In addition, two or more positive questionnaire responses constituted a significantly independent factor for non‐hypogonadal states (OR = 0.886; 95% CI = 0.802–0.98; *p* = 0.018).

**Table 4:** Multivariate analyses of factors associated with FT < 8.5 pg/mL.

*N* = 1609	Odds ratio	95% CI	*p*‐value
Age	1.065	1.052–1.079	**<0.01**
BMI	1.008	0.97–1.046	0.693
Number of positive (“yes”) questionnaire responses is >2			
	0.886	0.802–0.980	**0.018**
Dyslipidemia, yes	2.039	1.558–2.668	**<0.01**
Hypertension, yes	3.489	2.728–4.462	**<0.01**
Type‐2 diabetes, yes	3.035	2.296–4.01	**<0.01**

*Note*: Bold values indicate *p* < 0.05.

Abbreviations: BMI, body mass index; CI, confidence interval; FT, free testosterone.

## 4 DISCUSSION

This study suggested that belonging to athletic clubs during many periods from school age to the present is related to hypogonadism states in middle‐aged Japanese patients diagnosed later in their lives. This was the largest research in the literature that demonstrated that athletic affiliation exercise habits constitute an independent factor for the LOH syndrome within the Japanese population. Sixty‐four percent (135/211) of those who answered that they were currently in an athletic club provided three or more positive (“yes”) questionnaire responses (Figure 2). This indicates that the majority of those who currently belong to an athletic club have been members of a sports club since their school days, thus suggesting that they have continued the habits of exercising since childhood. In this study, provision of two or more positive questionnaire responses of the questionnaire was a significant and independent factor for avoiding hypogonadal states in multivariate analysis. Implementing exercise habits from childhood or adolescence is considered to be related to high‐testosterone levels independently of diabetes, age, and hypertension. The results of each of the exercise habits questions showed no significant differences in testosterone levels for either “yes” or “no” answers (Table 2). This also could support the aforementioned conclusions.

Several research studies that have focused on short‐term exercise and testosterone have been reported.^14^, ^18^ They have suggested that regular 12‐ and 6‐week acute aerobic exercise increases circulating levels of testosterone in healthy young and older men. These previous studies have implied that bouts of aerobic exercise, particularly high‐intensity aerobic exercise, increase serum testosterone levels in men. However, it could not be ruled out that high‐intensity exercise only temporarily increases testosterone levels. None of the trials were able to evaluate the association between exercise habits and long‐term testosterone levels. This study discusses the association between exercise routine from childhood, which we believe is noteworthy because (to our knowledge) few relevant reports have been published.

Not only high‐intensity exercise but also low‐intensity sport could make a positive impact on the development in testosterone.^19^ In our study, exercise habits were only assessed in terms of membership of a sports club, which may include subjects who only habitually engage in low‐intensity exercise. Therefore, the aforementioned research further support our finding that longer involvement in sports during school years is associated with higher testosterone levels, even in those who were not members of a high‐intensity sports club. Furthermore, intensive exercise cause carbohydrate rather than lipid oxidation, are orexigenic, and could paradoxically result in weight increase. Moreover, participants have worse compliance to intensive exercise regimens.^20^ This is why, especially in obese participants, a low‐intensity stamina exercise focused on maximal muscular lipid oxidation may be recommended.^21^ In view of the above, exercise habits may be more important for testosterone development than exercise intensity.

García‐Hermoso et al., have suggested that cardiorespiratory fitness (CRF) levels during youth and their improvement may be associated with a lower risk of developing obesity and cardiometabolic disease later in life.^22^ They also concluded that early intervention and supporting prevention strategies that promote CRF may help children maintain or achieve a healthy status and thus help circumvent many future health problems because the origins of cardiometabolic disease begin early in childhood and cardiometabolic disease risk factors continued from early childhood to adulthood. In view of the relationship between testosterone values and metabolic syndrome, their research also suggests that long‐term rather than short‐term exercise habits may affect future testosterone levels. Sports club activity in childhood may possibly affect future testosterone levels, especially during secondary sexual characteristics, which may in turn affect obesity and hypogonadism. Panagiota et al., have even implicated that in young male athletes, multiple modes of exercise could lead to an elevation of cortisol and testosterone, thus maximizing the beneficial effects on growth and development, when exercise is performed in the evening hours.^23^ In terms of childhood and evening exercise, belonging to a school‐age sports club is just perfect. Our study supports these research.

BMI and waist circumference were negatively correlated with testosterone levels, although BMI was not associated with low testosterone levels in this study.^24^ Some Japanese studies have found no association between BMI and low‐testosterone, similar to this study.^9^ The prevalence of obesity and overweight in Japanese adults is rather low in international comparisons^25^ and could explain why BMI was not a significant factor in this study.

This study is associated with some limitations that are worth noting. First of all, this questionnaire was filled in by the patients themselves, and therefore the reliability of the results could not be guaranteed. Second, the patients only confirmed their affiliations with athletic clubs from their school days, and the types of daily physical activities were unclear owing to the many types of athletic clubs. The “exercise habit questionnaire” in this study just examined whether and when the participants were athletic club members. The participants belonging to an athletic club may not directly indicate participants' “exercise habits” or “exercise routine” during young age. The amount of time spent in physical activity during their school years is also unknown. Kawaguchi et al. examined the association between group physical activity and exercise adherence among elderly community‐dwelling individuals who participated a community exercise club.^26^ The results showed that group program participants tended to participate in the exercise program more continuously than non‐participants (Prevalence ratio = 3.63; 95% CI: 1.98–6.65, *p* < 0.01). There was also a significant positive correlation between group program participation and exercise adherence for both women (8.08 [1.94–33.56], *p* < 0.01) and men (2.84 [1.39–5.78], *p* < 0.01). They concluded that this result suggested that group physical activity programs grow social interaction among participants and encouraged exercise persistence. Burke et al. also concludes that physical activity is more likely to be sustained in a group setting for elderly members as well as young adolescents than alone.^27^ The activities in an athletic club are group program itself, and these findings propose that belonging to an athletic club could be highly related to exercise habits. However, it does not directly reflect or be related to the actual “exercise habits” or “group exercise habits” in the past or now as well. Even in the recent study, there is an insufficiency of evidence/logic/references to confirm the claim that athletic club affiliation is related to different kinds of physical activity levels, exercise types, or habits. Therefore, it is considerable to refer to the conclusion of the current study that “athletic club affiliation is related to future serum FT levels” does not necessarily indicate that exercise habits from school age are associated with future testosterone.

We believe that a large and long‐term prospective cohort study is needed to determine what type of intensity of exercise (implemented from a young age) has a long‐term effect on future testosterone levels.

In addition, the examination of serum FT values using radioimmunoassay is generally unreliable according to the guidelines from the European Academy of Andrology,^3^ the American Urological Association.^28^ Hence, the British Society for Sexual Medicine currently recommends the usage of serum total testosterone (TT) values to determine LOH syndrome.^29^ Furthermore, the European Academy of Andrology has proposed to calculate FT values based on TT, albumin values, and sex hormone‐binding globulin.^3^ However, in a huge Japanese epidemiological research worked out, not serum TT values but FT levels was significantly correlated with age.^2^ This finding may be due to racial differences between Caucasians and Asians. Iwamoto et al. additionally reported a favorable correlation (*R* ^2^ = 0.4238) between analog and calculated FT values determined by radioimmunoassay.^2^ These high correlations have also been corroborated in another Japanese research.^30^ Therefore, Japanese guidelines recommend the usage of serum FT values for the diagnosis of LOH. Only single type of radioimmunoassay devise for FT measurement was allowed by the Japanese Ministry of Health, Labor and Welfare between 2007 and 2009 (during the period our participants examined serum FT). Moreover, this devise was abandoned and is no longer available.

On the other hand, testosterone measurements necessitate twice performance on separate days,^3^ but only single FT measurements was conducted at baseline in our study. In addition, testosterone levels in seminal plasma have recently been reported to reflect spermatogenesis in the testis.^31^ However, the relationship and differences between serum and seminal plasma testosterone values are unknown and little study has been conducted. It is also difficult to refer to the significance of seminal plasma testosterone measurements because it is unclear whether low plasma testosterone represents the same pathogenesis as testosterone deficiency that has been reported so far.^3^ Therefore, we believe that prospective studies on the association between exercise habits and future serum testosterone levels as well as plasma testosterone levels should be worthy of consideration.

Finally, there are many confounding factors in the studies about testosterone level that may affect their assessments and evaluation of the subjects (e.g., duration of marriage, sexual behavior, multiple illness, genitourinary diseases, BMI, lifestyle element such as smoking, alcohol, and exercise and many other confounders). However, this study did not take these confounding factors into account in the results.

Hence, further prospective research with precise control of confounders, including multiple FT calculations and a number of evaluations at baseline, are necessary to prove the correlation between exercise habits from school age and the future hypogonadism among senior Japanese patients.

In conclusion, membership in athletic clubs during many periods from school age to the present is significantly associated with hypogonadism states in middle‐ and old‐aged Japanese patients. Most of those who were currently in athletic teams have been in athletic teams since they were in school. This was the first and largest study to demonstrate that athletic affiliation exercise habits were an independent factor for LOH syndrome within the Japanese population. This outcome proposes that athletic affiliation from childhood may affect future testosterone levels.

## AUTHOR CONTRIBUTIONS

**Yuki Kato**: Conceptualization; investigation; project administration; writing—original draft. **Kazuyoshi Shigehara**: Conceptualization; data curation; formal analysis; investigation; project administration; writing—review and editing. **Tomomi Nakagawa**: Data curation; supervision. **Shohei Kawaguchi**: Investigation; methodology; project administration; supervision. **Kouji Izumi**: Supervision; writing—review and editing. **Yoshifumi Kadono**: Supervision; writing—review and editing. **Atsushi Mizokami**: Project administration; software; supervision; writing—review and editing.

## CONFLICT OF INTEREST STATEMENT

The authors declare no conflict of interest.

## TRANSPARENCY STATEMENT

The lead author Yuki Kato affirms that this manuscript is an honest, accurate, and transparent account of the study being reported; that no important aspects of the study have been omitted; and that any discrepancies from the study as planned (and, if relevant, registered) have been explained.

## Supporting information

Supporting information.

## Data Availability

The data that support the findings of this study are available on request from the corresponding author. The data are not publicly available due to privacy or ethical restrictions. All authors have read and approved the final version of the manuscript. The corresponding author will made the data available upon reasonable request in this study and takes complete responsibility for the integrity of the data and the accuracy of the data analysis.
